# A case of acute tubulointerstitial nephritis following administration of the Oxford-AstraZeneca COVID-19 vaccine: a case report

**DOI:** 10.1186/s12882-023-03089-2

**Published:** 2023-03-14

**Authors:** Samuel B. M. Williams, Stephen D. J. Holwill, Rhian L. Clissold, Coralie Bingham

**Affiliations:** 1 Royal Devon and Exeter NHS Foundation Trust, Barrack Road, Exeter, EX2 5DW UK; 2 Taunton and Somerset NHS Foundation Trust, Musgrove Park Hospital, Musgrove Park, Taunton, TA1 5DA UK

**Keywords:** Acute kidney injury, Tubulointerstitial nephritis, COVID-19, Vaccination, Case report

## Abstract

**Background:**

More than 4 billion doses of the Coronavirus disease (COVID-19) vaccine have been administered worldwide but the relationship between the different vaccines and the development of renal disease is unknown. We present a case of tubulointerstitial nephritis following administration of the Oxford-AstraZeneca COVID-19 vaccine.

**Case presentation:**

A previously fit and well 51-year-old female presented on 27th May 2021 with a one-month history of weight loss, fatigue, nausea, and a metallic taste. She had an acute kidney injury with a creatinine of 484 umol/L. She was on no regular medications and denied taking any over-the-counter or alternative medicines. She had received her first dose of the Oxford-AstraZeneca vaccine on 23rd March 2021 and her second dose on 20th May 2021. A renal biopsy was performed the following day. The 19 glomeruli appeared normal to light microscopy but the tubulointerstitial compartment contained a dense inflammatory infiltrate including many eosinophils. There was widespread acute tubular injury with tubulitis, but no established or longstanding atrophy. A diagnosis was made of an acute tubulointerstitial nephritis. She was commenced on oral prednisolone and her renal function improved. She did not require renal replacement therapy at any time.

**Conclusions:**

To our knowledge, this was the first described case of acute tubulointerstitial nephritis following administration of the Oxford-AstraZeneca COVID-19 vaccine, although a number of cases have emerged more recently. In our case the patient was very fit and well, had no previous past medical history and had not taken any recent prescribed, over-the-counter or alternative medications. The absence of these provoking factors in our case makes the vaccine the most likely explanation for the development of tubulointerstitial nephritis although the pathophysiology behind this remains unknown. Given the unprecedented number of vaccinations being delivered around the world, nephrologists should be aware of this possible link although more research into the topic is required.

## Background

More than 4 billion doses of the Coronavirus disease (COVID-19) vaccine have been administered worldwide since the beginning of the vaccination programme. The relationship between the different COVID-19 vaccines and the development of renal disease is currently unknown. However, there are emerging reports of the vaccines being linked with the development of de novo and recurrent glomerular disease. A recent review article summarised several cases where patients had developed new or relapsing glomerulonephritis following administration of the COVID-19 vaccine [1].

The incidence of tubulointerstitial nephritis following administration of the COVID-19 vaccine is less widely reported although several cases have started to emerge within the literature [2–6].

Acute tubulointerstitial nephritis is typified by the presence of inflammation and oedema within the renal interstitium and is associated with acute renal injury. The clinical features are non-specific and include arthralgias, fever, a maculopapular rash and eosinophilia. Many cases present with no symptoms at all and therefore the condition is thought to be under-diagnosed by clinicians [7]. Drug-induced acute tubulointerstitial nephritis is by far the most common cause, representing 60–70% of cases [8]. Other causes include systemic diseases such as lupus or sarcoidosis, infectious causes including legionella and CMV and idiopathic causes such as tubulointerstitial nephritis with associated uveitis [7].

We present a case of tubulointerstitial nephritis in a patient with no previous history of renal disease following the second dose of the Oxford-AstraZeneca COVID-19 vaccine. Importantly, she had a very minimal past medical history and had taken no recent prescribed or over-the-counter medications.

## Case presentation

A previously fit and well 51-year-old female was referred to our renal team on 27th May 2021 with a presumed acute kidney injury (creatinine of 484 umol/L). She had never had her renal function checked previously. This was on a background of a one-month history of feeling non-specifically unwell. She gave a history of weight loss (6 kg), fatigue, nausea and a metallic taste. She was on no regular medications and denied taking any over-the-counter or alternative medicines. She denied any illicit drug use. She had received her first dose of the Oxford-AstraZeneca vaccine on 23rd March 2021 and her second dose on 20th May 2021. Her physical examination was unremarkable. Specifically, she had no signs or symptoms of uveitis although a formal ophthalmological examination was not sought.

**Fig. 1:**
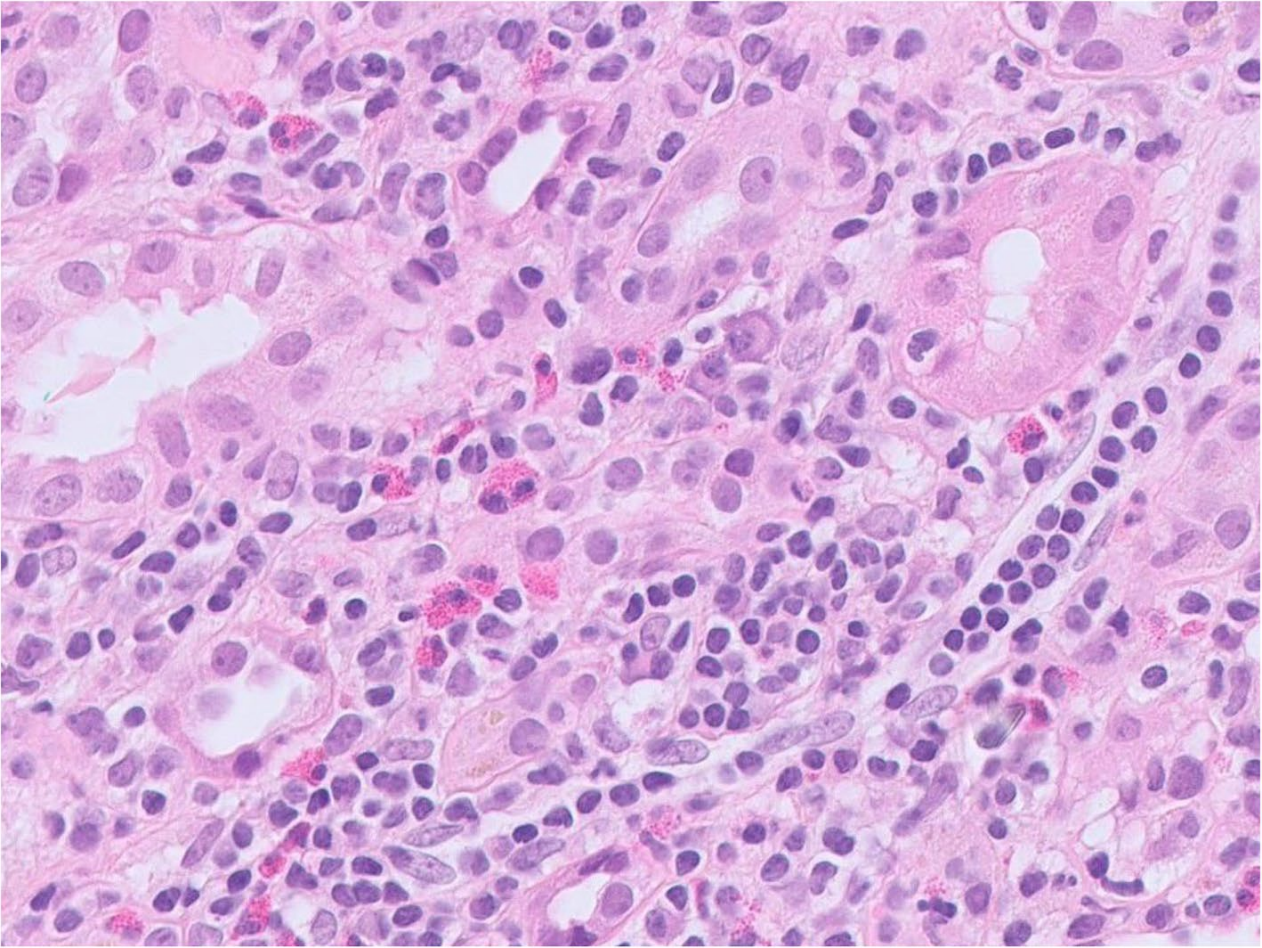
Renal biopsy demonstrating a diffuse cellular infiltrate within the interstitium with inflammatory cells including eosinophils and lymphocytes (hematoxylin and eosin stain)

Her blood pressure at presentation was 145/81. Her urinalysis showed 1+ protein and was positive for blood. The urine protein:creatinine ratio was 50 mg/mmol. Her CRP was 77, albumin 34 g/l and eosinophils were within the normal range at 0.16 10*9/L. Her acute renal screen was unremarkable; ANCA and Anti-nuclear antibody (ANA) were negative and her complement screen showed a mildly raised C3 of 1.89 g/L. She underwent a renal biopsy the following day that showed a total of 19 glomeruli, none were sclerosed. The glomeruli appeared normal to light microscopy. The tubulointerstitial compartment contained a dense inflammatory infiltrate including many eosinophils (Fig. 1). There was widespread acute tubular injury with tubulitis, but no established or longstanding atrophy. Only one small artery was seen and it appeared normal. Immunofluorescence staining was negative for Immunoglobulin G (IgG), Immunoglobulin A (IgA), Immunoglobulin M (IgM), C3, C1q, kappa and lambda. A diagnosis was made of an acute tubulointerstitial nephritis.

She was commenced on 40 mg of oral prednisolone which is the standard starting dose in our local renal unit. Her renal function continued to deteriorate for 3 days to a maximum creatinine of 626 umol/L before starting to improve. She did not require renal replacement therapy at any time.

Her renal function continued to improve on a 3-month weaning course of oral prednisolone, which was stopped at the end of August 2021. Her creatinine improved to 104 umol/L on 16th September 2021 and her latest creatinine was 78 on 17th March 2022.

## Discussion and conclusions

To our knowledge, this was the first described case of acute tubulointerstitial nephritis following administration of the Oxford-AstraZeneca COVID-19 vaccine. One case of tubulointerstitial nephritis was reported after the administration of the Pfizer-BioNTech vaccine in a patient with pre-existing chronic kidney disease [9]. However, the patient in this case was a 78-year-old man with multiple comorbidities including hypertension and diabetes with nephropathy. Importantly, he was also taking several regular medications including allopurinol, metformin, vildagliptin, a statin and an ace-inhibitor.

A further case of tubulointerstitial nephritis was reported following administration of the first dose of the BioNTech vaccine [10]. A 44-year-old female presented with headache, fever, nausea and weakness 48 hours after receiving the vaccine. She required acute dialysis the following day and a subsequent biopsy detected extensive inflammation in the tubulointerstitial area with occasional eosinophils and preserved glomeruli. Creatine kinase, ANCA, ANA and complement levels were normal and she was diagnosed with vaccine induced tubulointerstitial nephritis. She responded to steroid treatment with complete renal recovery. This patient was not known to have any systemic disease but it is unclear whether she had taken any prescribed or over-the-counter medications in the preceding months. In addition, it is unusual to develop interstitial nephritis with such a significant acute kidney injury so soon after the offending drug is administered.

A number of case reports of tubulointerstitial nephritis have emerged more recently [2–6]. They have been associated with a number of different types of vaccine (including the Oxford-AstraZeneca) and although some of these cases were in comorbid patients taking multiple medications including proton pump inhibitors, other cases were in previously fit and well patients taking no regular medications, similar to the patient in our case.

Interestingly, acute tubulointerstitial nephritis has also been associated with the influenza vaccination in one case study [11]. However, in this case, the patient had also taken a non-steroidal anti-inflammatory just after the vaccination, which may provide an alternative explanation for his tubulointerstitial nephritis.

There have also been various case reports linking the vaccine with the development of de novo and relapsing glomerulonephritis [1].

Given that billions of people are being vaccinated around the world, it is plausible that the development of rare kidney disease is temporally associated with the COVID-19 vaccination but may be unrelated to the vaccine itself. It is very difficult to prove a causal relationship in all the above case studies. It is also conceivable that another ingredient or adjuvant within the vaccine preparation could cause a tubulointerstitial nephritis.

In our case the patient was very fit and well, had no previous past medical history and had not taken any recent prescribed, over-the-counter or alternative medications. The absence of these other provoking factors in our case makes the vaccine the most likely explanation for the development of tubulointerstitial nephritis. The timescale between receiving the vaccination and presenting with acute kidney injury also supports this theory although the pathophysiology behind this remains unknown. Given the unprecedented number of vaccinations being delivered around the world, nephrologists should be aware of this possible link although more research into the topic is required.

## Data Availability

Not applicable as no data was used in this article.

## Abbreviations

Covid-19: Coronavirus disease 2019
ANCA: Antineutrophil Cytoplasmic Antibody
IgA: Immunoglobulin A
ANA: Antinuclear antibody
IgM: Immunoglobulin M
IgG: Immunoglobulin G
mRNA: messenger RNA
